# Beyond the Cochrane review: Re‐evaluating anti‐amyloid therapy in Alzheimer's disease

**DOI:** 10.1002/alz.71769

**Published:** 2026-08-21

**Authors:** Megan M. Bernath, Jared Brosch, Andrew J. Saykin, Donna Wilcock, Diana Summanwar, Deanna R. Willis

**Affiliations:** ^1^ Department of Family Medicine Indiana University School of Medicine Indianapolis Indiana USA; ^2^ Indiana Alzheimer's Disease Research Center Indiana University School of Medicine Indianapolis Indiana USA; ^3^ Department of Neurology Indiana University School of Medicine Indianapolis Indiana USA; ^4^ Center for Neuroimaging, Department of Radiology and Imaging Sciences Indiana University School of Medicine Indianapolis Indiana USA; ^5^ Stark Neurosciences Research Institute Indiana University School of Medicine Indianapolis Indiana USA

**Keywords:** Alzheimer's disease, amyloid cascade hypothesis, amyloid beta, amyloid‐related imaging abnormalities (ARIA), anti‐amyloid therapy, Cochrane review, cognition, donanemab, lecanemab

## Abstract

The global burden of Alzheimer's disease (AD) is accelerating, with U.S. prevalence projected to reach nearly 13 million by 2060. Although the “amyloid cascade hypothesis” has long‐guided drug development, a 2026 Cochrane review challenged amyloid beta (Aβ) targets for therapy by citing insufficient clinical benefit and safety concerns. We highlight several methodological limitations in this study. The primary concern is a “dilution effect” caused by analyzing monomer‐selective antibodies alongside newer treatments that directly target toxic Aβ plaques. This statistical noise, compounded by a decade of failed studies, obscures the significant cognitive preservation achieved by U.S. Food and Drug Administration (FDA)–approved therapies, like lecanemab and donanemab. We contend that these cognitive outcomes are clinically meaningful, as dismissing them ignores the vital impact on preserved independence and reduced caregiver burden. We emphasize current stratified safety frameworks for amyloid‐related imaging abnormalities (ARIA) management. The path forward lies in an integrated framework combining Aβ clearance with novel, multi‐pathway therapies.

## Highlights

- Methodology limits a recent Cochrane review's conclusion on anti‐amyloid therapy.
- We emphasize current stratified safety frameworks for managing amyloid‐related imaging abnormalities (ARIA) complications.
- Ignoring anti‐amyloid therapy (AAT) progress devalues patient independence and vital caregiver relief.
- The field must embrace a synergistic, multi‐pathway future for Alzheimer's disease.

## 1 INTRODUCTION

Alzheimer's disease (AD) is a progressive neurodegenerative disorder characterized by cognitive decline and loss of functional independence in daily life. Demographic trends indicate a growing older adult population, driven by lower mortality rates and increased longevity.^1^ AD is currently the seventh leading cause of death. Projections indicate that the population of U.S. adults 65 years of age and older with AD will expand to ≈13 million by 2060.^2^

AD is pathologically marked by early buildup of amyloid beta (Aβ) in the brain. Aβ proteins originate as monomeric peptides, whose physiologic role remains uncertain, but may include neuroprotective functions. These peptides can aggregate into oligomers and plaques. This aggregation is widely considered among the earliest pathologic changes of AD and may occur up to 25 years before symptom onset.^3^ This pathology is followed by the appearance of neurofibrillary tau tangles, which are clinically associated with the start of symptoms.^4^ For decades, the “amyloid cascade hypothesis” has been a leading theory of AD pathogenesis.^5^ Accordingly, most agents studied to slow the clinical decline of AD have been monoclonal antibodies targeting Aβ.^6^ Prior to 2022, all potential treatments targeting Aβ had failed to show significant clinical outcomes, and drug effects on amyloid plaque burden varied substantially.^7^ Recent years have seen progress with the regulatory approval of anti‐amyloid therapies (AATs) that have reduced Aβ levels and slowed cognitive decline.^7^ Rigorous, evidence‐based reviews are vital to help guide researchers, clinicians, and patients navigate a rapidly evolving field.

A 2026 Cochrane review of AATs synthesized data from 17 clinical trials, encompassing over 20,000 participants with mild cognitive impairment or mild dementia.^8^ The authors concluded that drugs targeting Aβ proteins in the brain provided no clinically meaningful benefit in the symptomatic progression of AD, despite an observed reduction of brain amyloid plaques. Furthermore, the review highlighted safety concerns, specifically related to the prevalence of therapy‐induced amyloid‐related imaging abnormalities (ARIA). The review authors questioned the validity of the “amyloid hypothesis,” contending that the decoupling of plaque clearance from cognitive preservation necessitates a paradigm shift in Alzheimer's research. The article concluded that the current evidence does not justify the risk and costs of continued trials with AATs, advocating for the prioritization of other pathways.

## 2 METHODOLOGICAL CONSIDERATIONS

Clinical reviews are powerful tools for guiding the field, but they are dependent on sound methodology. Adhering to strict scientific rigor is essential to ensure that the resulting conclusions are actionable and avoid faulty assertions. Several methodological limitations affect the validity of this evidence review. First, the search criteria lacked sufficient stratification across AAT mechanistic classes. This review's eligibility criteria included outcomes from randomized controlled trials for “amyloid‐beta‐targeting monoclonal antibodies” with various mechanisms of action. However, the literature emphasizes that drug properties are determinants of clinical performance.^5^ For example, AATs have targeted a variety of Aβ forms, some of which have shown no clinical action in terms of amyloid removal (i.e., targeting soluble Aβ monomers). The first‐generation monoclonal antibodies solanezumab, bapineuzumab, and crenezumab predominantly targeted amyloid monomers and failed to reduce brain plaques or prevent cognitive decline.^9^, ^10^, ^11^ In contrast, AATs that target toxic oligomers, protofibrils, and plaques, including lecanemab, donanemab, aducanumab, and gantenerumab, can remove and/or prevent amyloid plaque formation. Some agents in this class have demonstrated statistically significant, although modest, clinical benefit.^12^, ^13^, ^14^ By pooling data from heterogeneous therapeutic candidate drugs, this review obscured potential therapeutic differences across classes of monoclonal antibodies, as well as between those from failed trials and those that met a priori endpoints leading to approval. A meta‐analysis performed by Avgerinoes et al. (2024) found that antibodies without monomer binding were associated with the most favorable clinical effects, supporting the notion that aggregate‐targeting confers both greater amyloid reduction and greater clinical signal.^15^ Ultimately, the methodological heterogeneity inherent in this review may have introduced sufficient statistical noise that limits interpretability of any discernible therapeutic effect.

Second, conclusions drawn from a decade of failed drug therapy trials introduce temporal confounding. Anti‐amyloid antibodies have only recently achieved some level of clinical efficacy, with two drugs achieving full U.S. Food and Drug Administration (FDA) approval.^7^ Combining trials may create a “dilution effect,” where the clinically meaningful efficacy of later agents (*n* = 2) is statistically masked by a legacy of historical failures (*n* = 15). In addition, this methodology masks the clinically significant effect of recent breakthrough agents by the statistical weight of historical findings. This strategy deprioritizes clinical and biological discovery and produces an overly simplified average of the past decade of work. It also potentially disincentivizes the publication of negative studies when they are used in this manner.

Finally, the authors primarily interpreted therapeutic efficacy through fixed‐timepoint estimates, most notably at the 18‐month mark, rather than longitudinal modeling. These restricted timelines fail to account for the longitudinal trajectories captured in a longer‐running, placebo‐controlled protocol. Given that AD progresses over decades, evaluating disease‐modifying therapies within a narrow therapeutic window omits the cumulative benefits that manifest at later timepoints. A longitudinal approach may increase the ability to detect disease‐modifying treatment effects, since the therapeutic benefit may compound over time.^16^ In support, gantenerumab showed no significant clinical effects in short term (2–3 years) removal of amyloid, but longer‐term (average 8 years) findings showed a potential delay in symptom onset and dementia progression.^17^ Gantenerumab findings highlight the importance of continued support for longitudinal, real‐world data that are now being captured in clinical practice.

## 3 ACCESSIBILITY AND FEASIBILITY BARRIERS TO ALZHEIMER'S AMYLOID‐TARGETING THERAPIES

Although AATs hold potential to change the trajectory of AD cognitive decline, there are substantial barriers to the applicability and generalizability of these therapies. One major barrier is the need for accurate and earlier diagnosis of AD. Current guidelines for study inclusion are biomarker‐driven, requiring access to amyloid positron emission tomography (PET) or cerebrospinal fluid (CSF), to diagnose AD. Patients in geographically isolated regions and lower socioeconomic classes will likely have difficulty accessing the resources required for AAT testing, monitoring, and infusion therapy.^7^, ^18^, ^19^ To this end, blood biomarker development is crucial for the number of cases that will require evaluation and for patient access and affordability.^20^

There are concerns that specialist and facility capacity could restrict access for treatment. Both FDA‐approved drugs are currently administered intravenously at infusion centers, and it has been modeled that ≈2.1 million individuals with early dementia would develop AD while waiting for testing and treatment.^21^ The recent emergence of subcutaneous lecanemab could reduce health system burden and add time savings and convenience for patients.^22^ Studies have shown that oral AATs did not achieve prespecified endpoints,^23^, ^24^, ^25^ but avenues for oral therapy targeting other pathways are currently being pursued in clinical trials.^26^ Medication cost and compliance are a concern, especially for patients on fixed budgets. Meta‐analyses suggest that the lecanemab discontinuation rate for any reason was 17%, but compliance should be closely monitored as AATs become more widespread.^27^ Lecanemab and donanemab have received traditional approval, which allows broader access through Medicare, although Medicare Advantage beneficiaries can have coverage gaps in their deductible and copayments that may result in unaffordable medication coverage.^28^ The Medicare landscape can also be regional, with Medicare intermediaries sometimes having different coverage administrative processes that could create challenges in some regions of the country.^29^ Overcoming these challenges will require continuous research, healthcare infrastructure adaptations, and supportive policies to ensure that AATs are integrated into standard care safely, effectively, and equitably.

The cost–benefit discussion of AATs is a debated topic. Studies that incorporate AAT costs, such as PET or CSF assays, physician visits, apolipoprotein E (*APOE*) ɛ4 testing, AAT infusions, and magnetic resonance imaging (MRI) scans, have concluded that AATs are not cost‐effective relative to standard of care.^30^ Other studies suggest that AAT treatment reduces medical costs and hours of care required from care partners, and improves quality of life.^31^ Regardless, any potential benefit of anti‐amyloid therapy is confined to a highly selective patient subpopulation due to strict inclusion and exclusion criteria.^32^ It has been suggested that less than 90% of patients may be ineligible for AAT under current guidelines, thereby severely limiting its broader clinical utility in real‐world cohorts.^33^ Although AAT could reduce the overall societal and personal economic burdens of AD, the immediate cost of pharmaceutical agents and monitoring pose a significant barrier to access.^30^, ^34^ There is value to be gained with developing more affordable, next‐generation treatments that have a larger impact on slowing cognitive decline.

## 4 ANTI‐AMYLOID THERAPY SAFETY PROFILE

Current debate focuses on whether the clinical benefits of AATs are sufficient to justify their safety profiles, particularly concerning treatment‐emergent adverse events. Amyloid‐related imaging abnormalities of edema (ARIA‐E) and hemorrhages (ARIA‐H) are two vascular abnormalities known to occur spontaneously in AD and following Aβ antibody treatment. ARIA‐E and ARIA‐H have been observed in multiple Aβ monoclonal antibody therapy trials.^11^, ^35^ Although the exact mechanism remains unclear, ARIA is thought to be triggered by the rapid mobilization of antibody‐mediated Aβ aggregates from brain tissue and vessel walls. This clearance compromises vascular integrity, causing parenchymal fluid extravasation that is exacerbated by amyloid‐induced cerebrovascular weakening and underlying cerebral amyloid angiopathy.^36^ ARIA is frequently temporary and asymptomatic, though routine MRI monitoring is essential given its potential to cause severe complications.^37^ Symptoms can include headache, confusion, vomiting, seizures, stroke‐like symptoms, and, in rare cases, death.^38^ ARIA management varies based on symptomology. Patients with radiographically mild and asymptomatic ARIA can continue to receive treatment with close monitoring and dose titration, whereas patients with more severe findings may require treatment discontinuation or temporary suspension of therapy until ARIA resolution is achieved.^39^

The Cochrane review suggested that the clinical impact of AATs may not substantially offset the risks and the intensive monitoring required for treatment‐emergent adverse events. Their study reported a small increase in ARIA‐E occurrence in individuals receiving AAT compared to placebo or no therapy, citing 107 events per 1000 participants. The clinical utility of AAT is inextricably linked to the management of ARIA‐E and ARIA‐H. The FDA protocols approved a new titration dosing schedule for AATs to reduce ARIA risk.^40^, ^41^, ^42^ The FDA also introduced recommendations for ARIA monitoring when taking high‐risk medications, such as antiplatelet or anticoagulant drugs, and for carriers of the *APOE* ε4 allele.^43^ The *APOE* ε4 allele represents the most significant genetic risk factor for late‐onset AD, and some centers defer treatment or use more strict imaging criteria for patients who are homozygous for the ε4 allele.^44^ Beyond baseline eligibility, current research is focused on medication use, strict blood pressure management, and baseline white matter hyperintensity burden on brain MRI to minimize the likelihood and severity of ARIA events.^43^ For example, studies show that individuals with mean arterial pressures >93 mmHg may be associated with marginally high ARIA‐E risk and that the use of antihypertensives may decrease risk of ARIA‐E.^45^ Although protocols for the detection, monitoring, and management of ARIA are being refined to ensure clinical safety, it is important to consider the barriers to therapy that emerge with further restrictions.^46^ Careful consideration of the risk–benefit profile of emerging therapeutics will be instrumental in maximizing drug safety, tracking the long‐term clinical sequelae of ARIA, and providing access to the general population.

## 5 COGNITIVE OUTCOMES AND CLINICAL IMPLICATIONS OF AATs

The authors of the Cochrane review reported that the absolute effects of AAT on cognitive decline were “absent or trivial,” falling well below threshold for a clinically important difference. International experts in the care of patients and research in AD have developed multi‐perspective consensus recommendations, informed by patient and caregiver input to guide deeper understanding of meaningful clinical benefit. The Clinical Dementia Rating Sum of Boxes (CDR‐SB) and the Integrated Alzheimer's Disease Rating Scale (iADRS) are widely accepted as capturing changes in function and cognition that are meaningful to patients and their caregivers.^47^ Changes in these instruments have been associated with measures of patient reported quality of life and caregiver burden.^48^ These instruments, which assess memory and autonomy in daily activities, have been commonly used as primary clinical outcome assessments in AAT clinical trials. The CLARITY‐AD Phase 3 trial of lecanemab utilized the CDR‐SB scoring system to measure patient cognition and function. This study showed a 27% slowing of functional decline (CDR‐SB difference of −0.45) over 18 months, with all primary and secondary endpoints statistically significant (*p* < 0.001).^14^ Donanemab in the TRAILBLAZER‐ALZ 2 Phase 3 trial demonstrated a 29% slowing of functional decline (CDR‐SB difference of −0.67) over 18 months.^13^ Further analysis of iADRS outcomes demonstrated a donanemab impact on episodic memory and memory‐dependent activities of daily living.^49^ These findings, although clinically modest, mark an advancement over prior therapies and deliver clinically meaningful patient benefits, a perspective explicitly supported by the FDA committee.^50^, ^51^ Precedents in oncology underscore the value of modest clinical extensions, where life‐prolonging therapies often achieve regulatory approval with a median overall survival benefit of merely 2 to 4 months.^52^ For those living with the progression of AD, even a marginal deceleration of symptoms can yield a potentially meaningful impact, preserving the autonomy and personal dignity that define quality of life.^7^

Amyloid removal has shown to be impactful in individuals who are earlier in the disease process.^17^ ^,^ ^53^ A natural extension of these studies is to treat individuals who are “pre‐symptomatic,” that is, those individuals with amyloid plaque, low to absent neurofibrillary tau, and no symptoms. This novel use of AATs may be the ideal time to treat underlying amyloid pathology and is being pursued in the National Institutes of Health sponsored AHEAD 3‐45 trial, the Eli Lilly sponsored study Trailblazer ALZ3, and the recently announced Roche sponsored PrevenTRON studies.^54^, ^55^ The Alzheimer's Prevention Initiative (API) studied the use of the AAT, crenezumab, in cognitively unimpaired presenilin‐1 (*PSEN1*) autosomal‐dominant mutation carriers and non‐carriers in cognitive decline. This study demonstrated that crenezumab provided no significant benefit toward memory preservation or amyloid plaque reduction, indicating that robust clearance of fibrillar amyloid may be a prerequisite for clinical efficacy in patients with elevated amyloid burden.^56^ As previously mentioned, preliminary data in the Dominantly Inherited Alzheimer's Network Treatment Unit (DIAN‐TU), using gantenerumab showed delay to symptom onset in a small group of individuals monitored over an average of 8 years.^17^ These agents represent an important platform to build on. Rather than discarding current progress, we must pivot toward strategies that enhance efficacy. This includes exploring combination therapies and targeting the upstream pathways responsible for initial amyloid accumulation.^57^

## 6 SYNERGISTIC MULTI‐MECHANISM PATHWAY INVESTIGATION WITH AATs

The review authors recommended that funding resources be funneled into other pathways of interest. We agree that the field should continue to understand other potential mechanisms for AD that could synergistically improve cognitive function in patients with AD.^57^ Abandoning the amyloid hypothesis in favor of alternative theories trades one isolated mechanism for another. Instead, we should continue to advance our understanding of amyloid within the broader context of a multi‐mechanism disease landscape. It is important to note that in January 2026, there were over 192 clinical trials, with 158 novel drugs addressing 18 disease processes for AD.^26^ Phase 3 clinical trials have and are being pursued in other potential pathways in AD, such as the neurotoxic proteins tau and alpha‐synuclein, inflammation, metabolism, vasculature, neurogenesis, cell death, and the gut–brain axis, among other pathways.^58^ For example, the Alzheimer's Tau Platform is evaluating the safety and efficacy of tau‐directed therapies, either alone or combined with donanemab in the late preclinical or early prodromal stages of AD (NCT07167966). A second example is the PREVENTABLE study, conducted in individuals without dementia to demonstrate the benefit of atorvastatin for reducing death, dementia, and disability (NCT04262206). Finally, the established genomic link between the immune system and AD has driven the development of immunomodulatory therapeutics and vaccines that target specific immune cells and molecular mediators.^59^ These therapies modify disease progression through pathways independent of amyloid removal and are highly likely to provide additive clinical benefits when combined with approved AATs.

Alternative mechanisms have been identified and targeted with therapeutic agents for years. These pathways have been investigated extensively, but many late‐phase alternatives have not demonstrated clear clinical benefit. Unlike the recent successes in AATs, these diverse mechanistic approaches have not consistently demonstrated disease‐modifying effects in late‐phase trials. Recent high‐profile investigations into glucagon‐like peptide‐1 (GLP‐1) receptor agonists, such as semaglutide (EVOKE trials), sought to mitigate neuroinflammation and improve neuronal metabolism. Despite success in metabolic health, these have yet to yield clinically relevant cognitive results in AD.^60^ The close alignment between tau tangles and clinical symptoms led to the long‐held hypothesis that modifying tau aggregation would be the most effective pathway for AD, but this theory has yet to yield clinical success in late‐phase trials.^61^ Hypothesizing that vascular health drives AD, researchers tested statins and nonsteroidal anti‐inflammatory drugs (NSAIDs) to reduce cerebrovascular inflammation and cholesterol‐mediated damage. Large‐scale trials showed no significant benefit.^62^

Among the diverse therapeutic targets investigated in late‐phase clinical trials, AATs represent the only class of disease‐modifying therapy to date that have demonstrated a statistically significant impact on cognitive and functional decline. The fact that clearing amyloid provides a consistent, yet partial, slowing of the disease strongly suggests that clinical AD is driven by the dysfunction of multiple, intersecting biological pathways.^63^ Multi‐mechanism conditions are common in humans with oncologic, metabolic, and autoimmune conditions, frequently requiring multi‐mechanistic therapeutic approaches.^64^ Integration of therapeutics across mechanisms offers opportunities to build upon successes of AAT rather than discarding them. Experts in the field emphasize that, while add‐on therapy is essential to address the disease's multi‐pathway nature, its implementation requires overcoming substantial hurdles. To accelerate progress, the community must prioritize diverse aging‐related targets and leverage early‐phase trial data to identify synergistic drug therapy, particularly those that build upon the foundation of existing AAT.^65^

Finally, the pathways driving amyloid accumulation, and more broadly cognitive decline in older adults, are not likely to be same for all individuals—hence the need for a precision medicine approach to dementia. Genetic heterogeneity of *APOE* ε4 effects is already changing the way clinicians approach these treatments. This personalized approach can best be developed by building on current successes to extend impact and efficacy and ultimately establish preventative strategies.

## 7 CONCLUSION

Despite debate surrounding its absolute clinical impact, it is crucial to recognize that no historical trials have yet matched the clinical signal and biomarker success achieved by recent Aβ clearance therapies. For this reason, Aβ monoclonal antibodies maintain therapeutic relevance and merit clinical exploration, particularly in early‐stage AD and pre‐symptomatic, biomarker‐positive populations.^54^, ^55^ Although ongoing research aims to maximize the therapeutic potential of AATs, the benefit–risk profile of AATs remains a topic of debate. For families and individuals with AD, months of gained independence and cognition can translate into a meaningful impact on quality of life. The risks, such as ARIA, are becoming more manageable and better understood with monitoring. An evolving concern with real‐world, clinical integration of AATs is emerging due to limited system resources, drug and monitoring cost restrictions, and patient accessibility challenges. Altering disease progression remains the primary objective, but long‐term attention must also be placed on overcoming barriers to care.

Although current clinical trials underscore the significance of Aβ, it is essential to recognize that AD has a complex pathophysiology requiring continued investigation into multi‐pathway mechanisms. Rather than abandoning decades of foundational research, a more impactful approach would focus on identifying therapeutic pathways that drive and interact with Aβ plaque formation and the resulting downstream cascade of cognitive decline. AD clinical trials have historically targeted a diverse array of amyloid, metabolic, immunomodulatory, and tau‐centric pathways. Multi‐mechanistic therapies are supported by the finding that near‐complete removal of Aβ results in roughly 30% reduction of disease progression, implying that other therapeutic pathways could result in improved patient outcomes.^13^, ^14^ Emerging data, such as subgroup analyses from CLARITY‐AD, indicate that certain treated subpopulations, such as those with low‐to‐medium tau (TRAILBLAZER‐ALZ 2), experience minimal to no cognitive decline over several years.^66^ Consequently, establishing biomarkers to identify these optimal responders has become a high priority focus for many research institutions. Ultimate success in the field lies in integrating existing therapies with novel mechanisms that address the broader, multi‐pathway landscape of Alzheimer's progression in a manner tailored to the personalized biomarker profile of the individual, rather than in abandoning the first validated target for disease modification.

## CONFLICT OF INTEREST STATEMENT

M.M.B.: No related conflicts. J.R.B.: Has received institutional payments from Abbvie, Biogen, Eli Lilly, Athira, Alnylam, Eisai, and Washington University. He has received consulting fees from Eli Lilly, Abbvie, and Eisai. He has stock in Monument Biosciences. A.J.S.: Acknowledges support from multiple National Institutes of Health (NIH) grants (P30 AG010133, P30 AG072976, R01 AG019771, R01 AG057739, U19 AG024904, R01 LM013463, R01 AG068193, R01 AG092591, T32 AG071444, U01 AG068057, U01 AG072177, U19 AG074879, as well as U24 AG074855). He has also received in‐kind support from Avid Radiopharmaceuticals, a subsidiary of Eli Lilly (PET tracer precursor) and Gates Ventures, LLC and Sanofi (Proteomics panel assays on Indiana Alzheimer's Disease Research Center (IADRC) and Korean Brain Aging Study of the Early Diagnosis and Prediction of Alzheimer's Disease (KBASE) participants as part of the Global Neurodegeneration Proteomics Consortium), gift funds (GV) supporting technical contributions to the GRIP platform, and funding to IU by the Alzheimer's Drug Discovery Foundation's Diagnostics Accelerator (ADDF), and he has participated in Scientific Advisory Boards (Bayer Oncology, Bristol Myers Squibb, Eisai, New Amsterdam, Novo Nordisk, and Siemens Medical Solutions USA, Inc) and an Observational Study Monitoring Board (MESA, NIH NHLBI), as well as External Advisory Committees for multiple National Institute on Aging (NIA) grants. He also serves as Editor‐in‐Chief of *Brain Imaging and Behavior*, a Springer‐Nature Journal. D.M.W.: Is an Scientific Advisory Board (SAB) member and paid consultant for Vigil Neurosciences Inc., SynapsDx, Longeveron Inc., and Beren Therapeutics Inc. She also serves as Editor‐in‐Chief for the *Alzheimer's & Dementia Journal*. D.S.: No related conflicts. D.R.W.: Dr. Willis has grant funding through a subcontract with the American Academy of Family Physicians with American Academy of Family Physicians (AAFP) funding originating from Lilly Healthcare USA. She participates in the Medical Advisory Board for Voices Against Alzheimer's. She has had speaking engagements with the American Academy of Family Physicians with AAFP funding originating from Roche Diagnostics. She has had speaking engagements funded by UsAgainstAlzheimer's. Author disclosures are available in the Supporting Information.

## Supporting information



Supporting Information
